# Dominance style predicts differences in food retrieval strategies

**DOI:** 10.1038/s41598-021-82198-0

**Published:** 2021-02-01

**Authors:** Jose Luis Gomez-Melara, Rufino Acosta-Naranjo, Alba Castellano-Navarro, Victor Beltrán Francés, Alvaro Lopez Caicoya, Andrew J. J. MacIntosh, Risma Illa Maulany, Putu Oka Ngakan, Federica Amici

**Affiliations:** 1 Department of Social Anthropology, University of Seville, Seville, Spain; 2 Ethology and Animal Welfare Section, Universidad Cardenal Herrera-CEU, CEU Universities, Valencia, Spain; 3 Fundació Universitat de Girona, Innovació I Formació, Girona, Spain; 4 Department of Clinical Psychology and Psychobiology, Faculty of Psychology, University of Barcelona, Barcelona, Spain; 5 Primate Research Institute, Kyoto University, Aichi, Japan; 6 Forestry Department, Hasanuddin University, Makassar, Sulawesi Indonesia; 7 Behavioral Ecology Research Group, Institute of Biology, Faculty of Life Science, University of Leipzig, Leipzig, Germany; 8 Research Group Primate Behavioural Ecology, Department of Human Behavior, Ecology and Culture, Max-Planck Institute for Evolutionary Anthropology, Leipzig, Germany

**Keywords:** Animal behaviour, Anthropology

## Abstract

In several species, rank predicts access to food, and subordinates may need specific behavioural strategies to get a share of resources. This may be especially important in despotic species, where resources are strongly biased in favour of dominants and subordinates may more strongly rely on specific tactics to maximize food intake. Here, we compared three macaque species with an experimental set-up reproducing feeding competition contest. Following our predictions, more tolerant species mostly retrieved food in the presence of others and were less dependent on specific tactics. Contrarily, subordinates in more despotic species more likely collected food (1) when dominants could not see food or (2) were attacking others, (3) while “dissimulating”, or (4) “storing food”. Our study reveals that dominance styles reliably predict the probability of using specific food retrieval tactics and provides important insights on the social conditions that might have led to the emergence of tactical deception.

## Introduction

Across taxa, dominance rank usually predicts access to food: more dominant individuals can better access and monopolise resources, and often obtain more or better-quality food than subordinate group members^1–7^. If rank determines access to food, dominants and subordinates may rely on different strategies to maximize their food intake and thus their fitness. Dominants, for instance, may especially benefit from maintaining close proximity to food items that are more easily contested (e.g., because they are in spatial proximity to other group members; see Ref.^8–10^). In particular, by first approaching food items closer to possible competitors, dominants may displace potential competitors around unsecured food sources, and increase the chances of obtaining them^11–13^. Similarly, dominants may benefit from maintaining visual contact with the food resources available, to better monitor them and perhaps intimidate subordinates that may avoid retrieving food in the dominant’s view^14^.

In contrast, subordinates may use other strategies to increase their chances to obtain food. Firstly, they may avoid overlapping with dominants at feeding sites, by for instance using alternative and more peripheral food sources^11,15–17^. Secondly, subordinates may avoid retrieving food in the dominant’s view. At least in some species, subordinates may preferentially approach food sources that are not visible to dominant individuals^9,10,14,18–20^. Similarly, subordinates may “dissimulate” food retrieval, by e.g. collecting food while walking through the area, without slowing down and without extending the limbs, in a way that food retrieval is barely visible^18^. Finally, subordinates may delay food retrieval to a moment in which the risk of the dominant’s retaliation is lower, for instance because the dominant is already involved in an aggressive conflict with other individuals and is thus less likely to react against the subordinate retrieving food.

Moreover, while directly competing over food, both dominants and subordinates may try to maximize food intake by sequentially collecting several food items, without immediately consuming them. In some species, for instance, subordinates are more likely to store food in their cheek pouches when feeding competition is higher^21^. This strategy could allow them to collect food and move away to consume it in a safer place. However, such a strategy could also be used by dominants to quickly secure a larger number of food items without having to use aggressive behaviour toward competitors. In both cases, inhibitory skills are required, as individuals have to postpone immediate gains to obtain larger delayed ones^22^.

The importance of dominance rank for food acquisition, however, may differ across species. In non-human primates, species have different dominance styles, varying along a gradient from more despotic to more tolerant^23,24^. In more tolerant species, aggressive conflicts are expected to be less intense and characterized by higher counter-aggression rates, more undecided outcomes, higher reconciliation rate, less kin-bias in social interactions, and shallower dominance hierarchies, as compared to more despotic species^23,25^. In more tolerant species, therefore, the importance of rank for food acquisition may be minor, as subordinates may receive a higher share of resources or they may try to access food without a high risk of retaliation from more dominant group members^26,27^. In macaques, for instance, rank usually predicts access to food in more despotic species^11,15,28–32^, but results are contradictory in more tolerant species (for positive evidence^32,33^, for no clear effect^16,34^). Therefore, the need to undertake specific strategies in order to maximize food intake may be lower in more tolerant species, where tolerance over food may be higher.

In this study, we aimed to test whether differences in dominance style predict the occurrence of specific tactics to retrieve food and, in particular, whether individuals in more despotic species more frequently rely on these tactics, as conflicts over resources are generally more intense. We used an ecologically valid experimental set-up reproducing contest competition in feeding to test three free-ranging macaque groups with different dominance styles (more despotic Japanese macaques, *Macaca fuscata*, JM1; moderately tolerant Barbary macaques, *M. sylvanus,* BM3; and more tolerant moor macaques, *M. maura*, MM4; see Ref.^23,25^). Macaques are a monophyletic group with similar kinship and demographic structures across species^23,25,35^, and thus constitute an ideal model to test for the effect of dominance style while indirectly controlling for phylogeny. To compare their food retrieval strategies, we administered several experimental sessions in which we provided food to the group and monitored the identity and tactics used by every individual to retrieve food.

We predicted that, in the presence of other individuals, dominants in more despotic species would more often access food sources closer to other group members, in order to more effectively displace potential competitors (i.e. other group members who could otherwise collect the food) and thus secure access to more easily contested food items^11–13^ (Prediction dominant 1, hereafter P-Dom1). Moreover, we predicted that dominants in more despotic species would more often access food sources in places that allowed them to better monitor the remaining food (P-Dom2; Table 1). We further predicted that both dominants and subordinates in despotic species would more often collect food items without immediately consuming them: dominants to sequentially retrieve more pieces, and thus secure access to more food items (P-Dom3), and subordinates to move and eat it in another location, and thus avoid dominants’ retaliation (P-Sub1; Table 1). Finally, we predicted that subordinates in more despotic species would more often access food sources which are more peripheral (Prediction subordinate 1, P-Sub2) or not visible to the dominant (P-Sub3), and that they would more often dissimulate food retrieval (P-Sub4), or retrieve food when dominants are involved in aggressive interactions with other individuals (P-Sub5; Table 1).

**Table 1:** Predictions of our study, models used to test them, and whether they were confirmed.

Predictions	Models	Confirmed?
**In more despotic species, dominants more often…**		
P-Dom1. …access food closer to potential competitors	Dom1	No
P-Dom2. …access food allowing visual access to remaining food	Dom2	No
P-Dom3. …store food, to collect more pieces	DomSub1-2	**Yes**: JM1 > BM3, MM4
**In more despotic species, subordinates more often…**		
P-Sub1. …store food, to eat it in another location	DomSub1-2	**Yes**: JM1 > BM3, MM4
P-Sub2. …access peripheral food	Sub1	(in all species)
P-Sub3. …access food which is not visible to the dominant	Sub2	**Yes:** JM1 > MM4
P-Sub4. … “dissimulate” food retrieval	Sub3	**Yes:** JM1 > MM4
P-Sub5. …access food when dominants are attacking others	Sub4	**Yes:** JM1 > BM3 > MM4

## Results

In JM1 and BM3, individuals retrieved food when alone in the testing area in half of the trials (JM1: 50.1%, BM3: 50.5%). In contrast, MM4 mostly retrieved food in the presence of other group members (78.3%).

We first tested whether species predicted dominants’ probability of retrieving food closer to potential competitors (Model Dom1) or while maintaining visual access to most of the testing area (Model Dom2). However, both full-null model comparisons were not significant (GLMM: *χ*^2^ = 5.55, df = 2, *p* = 0.063; GLMM: *χ*^2^ = 1.07, df = 2, *p* = 0.586, respectively; Tables 1 and 2).

**Table 2:** Results of the models run, including estimates, standard errors (SE), confidence intervals (CIs), *z* values (*z*), likelihood ratio tests (LRT), degrees of freedom (df), and *p* values for each test and control predictor (in parentheses, the reference category).

Model	Estimate	SE	2.5% CI	97.5% CI	*z*	LRT	df	*P*
**DOM1: PROBABILITY THAT DOMINANTS COLLECT FOOD CLOSER TO COMPETITORS**								
Intercept	− 0.19	0.97	− 2.09	1.71	− 0.20	–	–	–
Species (JM1)	− 0.87	0.45	− 1.75	0.01	− 1.93	5.55	2	0.063
Species (MM4)	− 1.54	0.49	− 2.51	− 0.57	− 3.12			
*Rank*	− 1.98	1.05	− 4.04	0.08	− 1.88	3.14	1	0.076
*Session*	− 0.40	0.21	− 0.82	0.01	− 1.90	3.75	1	0.053
*Trial*	− 0.55	0.25	− 1.04	− 0.05	− 2.18	5.25	1	0.022
**Dom2: PROBABILITY THAT DOMINANTS COLLECT FOOD WHILE OBSERVING TESTING AREA**								
Intercept	1.00	0.79	− 0.55	2.55	1.26	–	–	–
Species (JM1)	− 0.55	0.52	− 1.56	0.46	− 1.06	1.07	2	0.586
Species (MM4)	− 0.43	0.46	− 1.33	0.47	− 0.94			
*Rank*	− 0.53	0.80	− 2.10	1.04	− 0.66	0.42	1	0.515
*Session*	0.08	0.09	− 0.11	0.26	0.83	0.69	1	0.405
*Trial*	− 0.28	0.10	− 0.47	− 0.08	− 2.73	7.57	1	0.006
**DomSub1: PROBABILITY TO TEMPORARILY STORE FOOD**								
Intercept	0.62	0.55	− 0.45	1.70	1.13	–	–	–
**Species** (JM1)	2.60	0.46	1.70	3.49	5.68	44.32	2	< 0.001*
**Species** (MM4)	− 0.31	0.42	− 1.12	0.51	− 0.74			
**Most dominant in the area**	− 1.71	0.27	− 2.24	− 1.17	− 6.24	43.19	1	**< **0.001*
*Rank*	− 2.29	0.79	− 3.83	− 0.75	− 2.91	7.75	1	0.005
*Age (juvenile)*	1.09	0.56	− 0.01	2.19	1.95	5.19	2	0.075
*Age (subadult)*	0.83	0.51	− 0.16	1.83	1.64			
*Session*	0.38	0.09	0.20	0.55	4.24	18.65	1	< 0.001
*Trial*	− 0.24	0.09	− 0.42	− 0.06	− 2.61	6.80	1	0.009
**DomSub2: PROBABILITY TO COLLECT MORE FOOD AFTER TEMPORARILY STORING IT**								
Intercept	− 1.57	0.51	− 2.56	− 0.58	− 3.10	–	–	–
**Species** (JM1)	0.72	0.32	0.10	1.34	2.29	13.08	2	0.001*
**Species** (MM4)	− 0.23	0.35	− 0.92	0.45	− 0.66			
**Most dominant in the area**	1.08	0.37	0.36	1.80	2.94	8.75	1	0.003*
*Rank*	0.58	0.75	− 0.89	2.04	0.77	0.60	1	0.438
*Age (juvenile)*	− 0.48	0.35	− 1.16	0.21	− 1.37	2.73	2	0.256
*Age (subadult)*	0.09	0.26	− 0.43	0.60	0.33			
*Session*	− 0.23	0.10	− 0.41	− 0.04	− 2.38	5.71	1	0.017
*Trial*	− 0.63	0.11	− 0.85	− 0.41	− 5.67	36.88	1	< 0.001
**Sub1: PROBABILITY TO RETRIEVE PERIPHERAL FOOD**								
Intercept	0.04	0.47	− 0.89	0.97	0.08	–	–	–
Species (JM1)	− 0.07	0.30	− 0.65	0.52	− 0.23	5.35	2	0.069
Species (MM4)	− 0.61	0.29	− 1.18	− 0.05	− 2.13			
**Being subordinate in the area**	0.90	0.21	0.50	1.31	4.39	20.33	1	< 0.001*
*Rank*	− 0.03	0.55	− 1.10	1.04	− 0.06	0.00	1	0.955
*Age (juvenile)*	0.01	0.37	− 0.71	0.74	0.03	0.00	2	0.999
*Age (subadult)*	− 0.01	0.34	− 0.68	0.66	− 0.02			
*Session*	0.10	0.06	− 0.02	0.22	1.62	2.64	1	0.104
*Trial*	− 0.08	0.06	− 0.20	0.04	− 1.26	1.58	1	0.209
**Sub2: PROBABILITY THAT SUBORDINATES COLLECT FOOD NOT VISIBLE TO DOMINANTS**								
Intercept	0.18	0.54	− 0.89	1.24	0.33	–	–	–
**Species** (JM1)	0.51	0.30	− 0.07	1.10	1.72	8.13	2	0.017*
**Species** (MM4)	− 0.24	0.30	− 0.82	0.34	− 0.80			
*Rank*	− 1.02	0.71	− 2.42	0.38	− 1.43	1.99	1	0.158
*Rank difference*	0.33	0.55	− 0.74	1.40	0.60	0.36	1	0.548
*Age (juvenile)*	0.38	0.32	− 0.24	1.00	1.19	1.57	2	0.456
*Age (subadult)*	− 0.01	0.29	− 0.59	0.56	− 0.04			
*Session*	− 0.11	0.08	− 0.26	0.04	− 1.41	1.99	1	0.159
*Trial*	0.58	0.09	0.41	0.75	6.66	48.97	1	< 0.001
**Sub3: PROBABILITY THAT SUBORDINATES COLLECT FOOD WHILE DISSIMULATING**								
Intercept	− 3.35	1.41	− 6.10	− 0.59	− 2.38	–	–	–
**Species** (JM1)	1.25	0.80	− 0.32	2.82	1.56	8.36	2	0.015*
**Species** (MM4)	− 0.19	0.88	− 1.91	1.53	− 0.21			
*Rank*	− 1.52	1.77	− 4.98	1.94	− 0.86	0.71	1	0.400
*Rank difference*	− 0.93	1.32	− 3.51	1.66	− 0.70	0.48	1	0.487
*Age (juvenile)*	0.41	0.69	− 0.95	1.76	0.59	3.61	2	0.165
*Age (subadult)*	0.98	0.49	0.02	1.94	2.00			
*Session*	0.53	0.20	0.13	0.93	2.59	7.28	1	0.007
*Trial*	− 0.13	0.18	− 0.49	0.23	− 0.71	0.52	1	0.469
**Sub4: PROBABILITY THAT SUBORDINATES COLLECT FOOD WHEN DOMINANTS ATTACK OTHERS**								
Intercept	− 2.77	0.92	− 457	− 0.96	− 3.01	–	−	–
**Species** (JM1)	1.02	0.37	0.30	1.74	2.76	36.73	2	< 0.001*
**Species** (MM4)	− 2.02	0.57	− 3.13	− 0.91	− 3.56			
*Rank*	− 0.29	1.25	− 2.75	2.16	− 0.24	0.05	1	0.816
*Rank difference*	1.56	0.86	− 0.13	3.25	1.81	3.44	1	0.064
*Age (juvenile)*	0.53	0.40	− 0.25	1.31	1.33	8.35	2	0.015
*Age (subadult)*	0.91	0.34	0.24	1.57	2.69			
*Session*	0.05	0.10	− 0.15	0.26	0.52	0.27	1	0.606
*Trial*	0.15	0.10	− 0.04	− 0.34	1.55	2.39	1	0.122

Significant test predictors are in bold, control predictors in italics. Session and trial numbers were *z*-transformed prior to analysis. All models included subject identity as random effect. The asterisks denote significant *p* values for the test predictors. All models had a binomial distribution.

**Figure 1:**
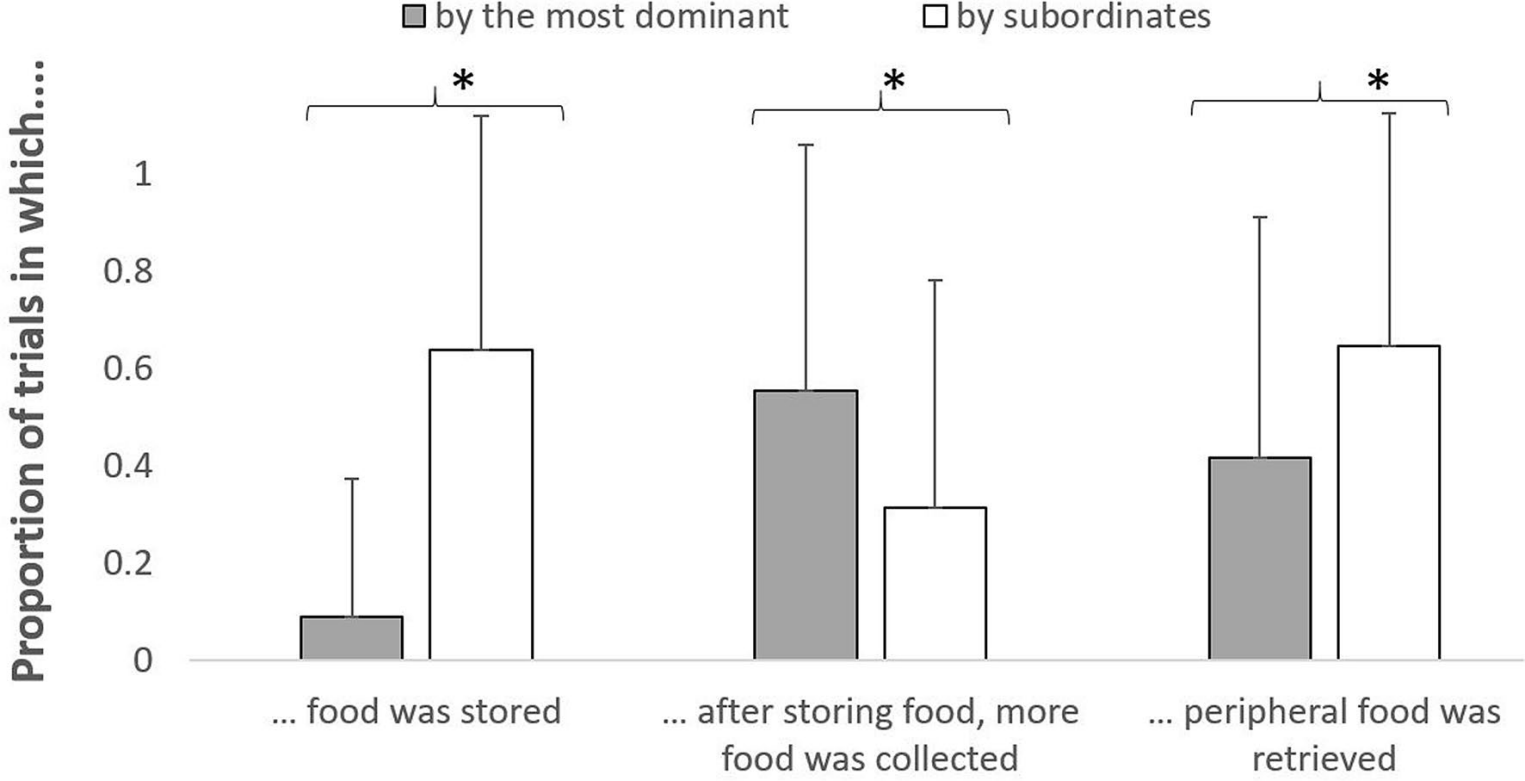
For dominants and subordinates separately, mean (+ SD) proportion of trials in which macaques stored food, in which they collected more food after storing one piece, and in which they retrieved peripheral food. Significant differences are marked with an asterisk.

**Figure 2:**
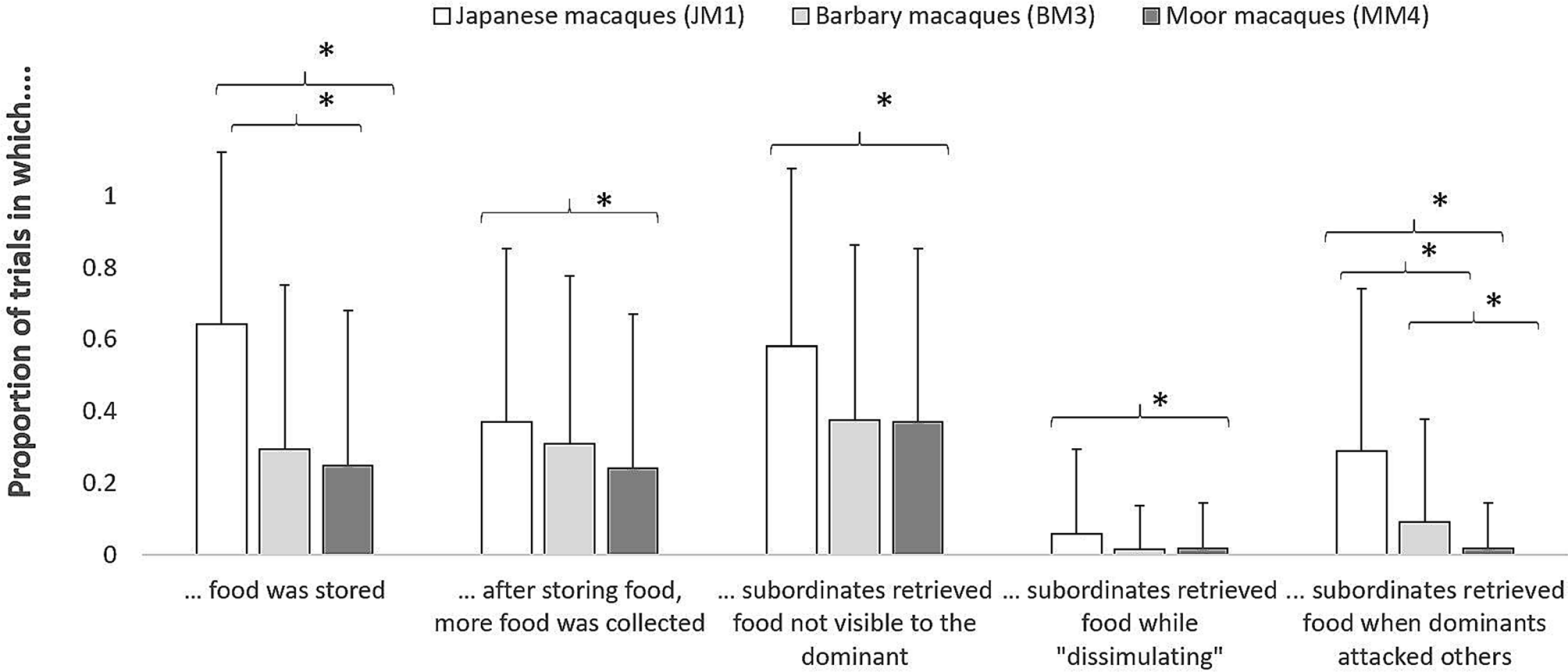
For each species separately, mean (+ SD) proportion of trials in which macaques stored food, in which they collected more food after storing one piece, in which they retrieved food which was not visible to the most dominant, while “dissimulating”, or when dominants were involved in aggressive interactions with other individuals. Significant differences between species are marked with an asterisk.

We then tested whether the probability of temporarily storing food was higher in subordinates, and differed across species (Model DomSub1, GLMM: *χ*^2^ = 90.89, df = 5, *p* < 0.001; Tables 1 and 2). Our results showed that subordinates were more likely to temporarily store food (Fig. 1), and so were JM1 (as compared to BM3 and MM4; both *p* < 0.001; Fig. 2). Moreover, the probability of collecting more food after storing it was higher in dominants (Fig. 1), and also differed across species (Model DomSub2, GLMM: *χ*^2^ = 24.85, df = 5, *p* < 0.001; Tables 1 and 2), being overall higher in JM1 than in MM4 (*p* = 0.001) and marginally BM3 (*p* = 0.058; Fig. 2).

We further tested whether the probability of collecting peripheral food was higher in subordinates, and differed across species (Model Sub1, GLMM: *χ*^2^ = 29.11, df = 5, *p* < 0.001). Our results showed that subordinates were more likely to retrieve peripheral food, and this was true across all species (Tables 1 and 2; Fig. 1). Moreover, the probability that subordinates collected food when dominants turned their back to the food varied across species (Model Sub2, GLMM: *χ*^2^ = 8.13 df = 2, *p* = 0.017; Tables 1 and 2), being higher in JM1 than in MM4 (*p* = 0.005; Fig. 2). Also, the probability that subordinates retrieved food by dissimulating differed across species (Model Sub3, GLMM: *χ*^2^ = 8.36, df = 2, *p* = 0.015; Tables 1 and 2), being higher in JM1 than in MM4 (*p* = 0.034; Fig. 2). Finally, the probability that subordinates retrieved food when dominants were involved in aggressive conflicts with other group members varied across species (Model Sub4, GLMM: *χ*^2^ = 36.73, df = 2, *p* < 0.001; Tables 1 and 2), being higher in JM1 than in BM3 (*p* = 0.016) and MM4 (*p* < 0.001), and also being higher in BM3 than MM4 (*p* = 0.001; Fig. 2).

## Discussion

Our study revealed inter-specific differences in food retrieval strategies, which were in line with their dominance styles and largely confirmed our predictions (Table 1). In all species, subordinates were more likely than dominants to feed on peripheral food and leave the testing area after storing food. However, individuals in more tolerant species (MM4) showed higher tolerance over food, mostly retrieving food in the presence of others, and less likely recurring to specific tactics to maximize food income.

In more despotic species (JM1 and to a lesser extent BM3), in contrast, dominants and subordinates had a higher probability to store food than in more tolerant species, and they did it to pursue different goals (i.e. dominants to retrieve additional pieces, subordinates to eat food in a safer location^21^). In more despotic species, subordinates were also more likely to collect food that was not visible to dominants, while dominants were involved in aggressive interactions with others, or by dissimulating. In contrast, dominants’ tactics only partially differed across species, with dominant JM1 being more likely to temporarily store food to collect more, but having a similar probability of retrieving food closer to potential competitors or allowing them better visual access to the remaining food, as compared to more tolerant species. The fact that dominants did not use these strategies, also in JM1, might however also depend on the fact that lower-ranking individuals could have often avoided direct competition over the same piece of food, especially when the rank difference with the dominants was large.

These results are in line with previous studies suggesting that several primate species can use complex behavioural strategies^36,37^ and even take into account conspecifics’ visual perspective when retrieving food in a competitive context^9,10,14,18^. However, our results importantly add to previous research by showing how inter-specific differences in dominance style reliably predict the probability that these tactics are used. Different species cope with contest competition in different ways, and when tolerance over food is low, individuals may be forced to rely on these strategies to maximize food intake. Clearly, none of these strategies necessarily requires complex cognition (e.g. understanding others’ mental states). Individuals may have simply learned to use these strategies through trial-and-error learning, by having obtained gains in the past during similar interactions^6,38^. Similarly, this study cannot disentangle whether individuals take into account what other group members see or what they are attentive to. However, reliance on these strategies implies at least efficient learning abilities, sensitivity to a wide range of subtle social cues and high flexibility in using them^14,36,39^.

Our study provides novel findings about food retrieval strategies in three species with different dominance style. The three study groups, however, also differed in terms of group size (i.e. JM1: 53, BM3: 19; and MM4: 33) and, clearly, socio-ecological conditions, so that more studies on more conspecific groups are needed to confirm our results. For instance, it is possible that larger group sizes (rather than more despotic dominant styles) increase competition over food and thus the probability that food behavioural strategies emerge. This explanation, however, is unlikely for two main reasons. First, in our experiment we controlled for group size by providing individuals with a quantity of food proportional to the number of individuals in the group. Second, and more importantly, if larger group sizes predicted a higher probability of using food retrieval strategies, BM3 should show the lowest probability of using them, having the lowest group size, while our results showed that MM4 had the lowest probability. Similarly, it is possible that differences in the provisioning rates of the study groups affected our results, as all study groups were partially provisioned with small quantities of food by humans. Although it is hard to exactly assess the impact of food provisioning in the three groups, BM3 were the only group being provisioned on a daily basis (i.e. as compared to JM1 being provisioned twice a week, and MM4 occasionally). Given that the probability of using behavioural strategies was intermediate for BM3 (although they relied on food provisioning more than the other study groups), it seems unlikely that the extent of food provisioning can explain our results.

Overall, our study clearly shows that subordinates (and to a lesser extent dominants) rely on different tactics to maximize their food intake in a competitive context, also taking into account the visual perspectives of potential competitors. More crucially, our study reveals that differences in the species dominance styles reliably predict the probability that these tactics are used. To our knowledge, this is the first evidence of a link between the species dominance styles and the specific feeding tactics they use and provides important insights on the social conditions that might have favoured the emergence of complex forms of tactical deception.

## Methods

### Ethics

All experimental protocols were approved by the ethics committees of the Kyoto University Wildlife Research Center and the City of Kushima Agency for Cultural Affairs in Japan, by the Kementarian Negara Riset dan Teknologi Republik in Indonesia (RISTEK), and by the Helping Hand Trust in Gibraltar. As the study was based on observations and all study groups are occasionally fed (see below), no additional ethical permits were required. The study was carried out in accordance with the national regulations of the countries where the study took place.

### Subjects

We studied 3 free-ranging groups of macaques, belonging to three different species with different dominance styles, sensu Thierry^25^. We included 53 despotic Japanese macaques (grade 1: JM1) on Koshima island (Japan), 19 moderately tolerant Barbary macaques (grade 3: BM3) on the Gibraltar Upper Rock Nature Reserve Rock of Gibraltar (United Kingdom), and 33 tolerant moor macaques (grade 4: MM4) on Sulawesi island (Indonesia). The three groups included females and males of different age classes and ranks (Table S1). In all groups, monkeys exploited natural food, but they were also partially provisioned with small quantities of fruit and vegetables by humans (for more details on their living conditions, see Supplementary Material).

### Behavioural observations

When conducting no experiments, we carried out observations on all adults and subadults in each group. We recorded all the dyadic agonistic encounters with a clear winner-loser outcome that we observed (JM1: *N* = 2116; BM3: *N* = 126; MM4: *N* = 346). We noted the identity of both the winner and the loser, and the day of the encounter^40^. We then assessed the dominance hierarchy with the Elo-method (EloRating package, version 0.43), obtaining individual scaled Elo-ranks for each member of the three groups (see Supplementary Material; Table S1). Furthermore, to ensure that the study groups really differed in terms of dominance style as expected from literature, we directly assessed the steepness of the dominance hierarchy in each study group (see Supplementary Material).

### Experimental task

The task took place in a flat area with little to no vegetation that was habitually frequented by the groups during foraging. We prepared a testing area consisting of a square of 4 × 4 m subdivided into other 16 identical (1 × 1 m) squares marked with stones or branches. The four more central squares were considered as the “central area”, in contrast to the 12 more peripheral squares. A session started when food was spread in the testing area and a monkey entered the area. We used high-value food (i.e. banana slices in Gibraltar and Sulawesi, sweet potatoes in Koshima due to dietary restrictions), with the number of pieces in each session being proportional to the number of adults in each group (i.e. number of individuals × 0.4). Whenever a monkey entered the testing area, the experimenter named the monkey and specified its location, to ensure that all individuals could be later identified from the videos. The session ended (1) when all the food pieces were eaten, (2) if monkeys did not eat any piece for 30 s, or (3) when there were no individuals in the testing area for 30 s. Overall, we administered 60 sessions per group, across up to 9 testing days (JM1: 6 days; BM3: 7 days; MM4: 9 days). All sessions were video-recorded and were separated by at least a 5-min interval.

### Coding

We considered a trial as any event in which a monkey retrieved a piece of food. In each trial, we coded from the videos the identity of the individual retrieving food (i.e. subject), its species, rank and age class, whether there were other individuals in the testing area, the session number and the trial number of the session. If there was at least another individual in the testing area, we further coded whether the individual retrieving food was the most dominant one in the testing area, the position of the food retrieved (central versus peripheral), whether the food was immediately eaten or rather stored in the hand/cheeks, and in case of storing the food, whether the subject subsequently collected more food pieces or rather moved from the place of retrieval before eating it. If the subject retrieving food was the most dominant in the testing area, we further coded the occurrence of dominant strategies, i.e. whether the food retrieved was the closest one to the other individuals (as this is the food that the dominant should first collect to avoid it being retrieved by others), and whether the dominant collected the food while having visual access to at least 2/3 of the testing area. Finally, if the subject retrieving food was *not* the most dominant in the testing area, we further coded the occurrence of subordinate strategies, i.e. whether the dominant could see the food retrieved (i.e. whether the dominant’s face was oriented toward the food, or up to 90° to its right or left), whether the dominant was involved in an aggressive conflict with another individual when the food was collected, and whether the subordinate collected the food while dissimulating food retrieval (i.e. walking through the testing area and retrieving food without stopping or slowing down). The first author watched and coded all the videos. The last author watched all the videos to check the identity of the individuals in all trials (agreement: 97% out of 2355 trials), and further coded 20% of all the trials as explained above (agreement: 96% of the 472 trials). All trials in which the first and last author disagreed were discussed and observed until reaching an agreement.

### Data analysis

In the models, we included all the trials in which the individuals retrieved food when at least two individuals were in the testing area. Analyses were conducted using generalized linear mixed models^41^ with the glmmTMB package (version 1.0.1^42^) in R (R Core Team, version 3.5.0). Firstly, we assessed whether the most dominant individuals in the area used different tactics to maximize their food intake, depending on the species (Predictions P-Dom1, P-Dom2). We entered one line for each trial in which the most dominant individual in the area retrieved the food, and tested whether species predicted dominants’ probability to retrieve food closer to potential competitors (Model Dom1), or while maintaining visual access to most of the testing area (Model Dom2). In both models, we controlled for subject’s rank, session and trial number, including subject identity as random effect. We did not control for subject’s age class, as all subjects in these trials were adults.

To assess inter-specific variation in food storing tactics (Predictions P-Dom3, P-Sub1), we entered one line for each trial (i.e. those in which the most dominant in the area retrieved the food, and those in which food was retrieved by more subordinate individuals) and tested whether being the most dominant individual in the area (in interaction with species) predicted whether subjects would temporarily store food (Model DomSub1). If so, we entered one line for each trial in which food was temporarily stored, and tested whether they would collect more pieces after storing food (or rather leave; Model DomSub2). In both models, we controlled for subject’s rank and age class, session and trial number, including subject as random effect.

Finally, we assessed the effect of species on the probability that subordinates used different tactics to retrieve food (Predictions P-Sub2 to P-Sub5). First, we entered one line for each trial (i.e. those in which the most dominant in the area retrieved the food, and those in which food was retrieved by more subordinate individuals), and tested whether being a subordinate individual in the area (in interaction with species) predicted whether food would be collected in the peripheral area (Model Sub1), when controlling for subject’s rank and age class, session and trial number, including subject as random effect. Furthermore, we entered one line for each trial in which food was retrieved by a subordinate in the area, and tested whether species predicted subordinates’ probability to retrieve food out of the dominants’ view (Model Sub2), while dissimulating (Model Sub3), or when dominants were involved in aggressive conflicts with others (Model Sub4), always including subject as random effect, and controlling for subject’s rank and age class, rank difference between dominant and subject (as the need to use these strategies might decrease with little rank difference), session and trial number.

All models were run with a binomial structure, previously *z-*transforming continuous predictors (i.e. session and trial number) to facilitate model convergence and interpretation of model coefficients. We used likelihood ratio tests^43^ to compare full models containing all predictors with null models containing only control predictors and random factors. When full models significantly differed from null models, likelihood ratio tests were conducted to obtain the *p* values for each test predictor via single-term deletion using the R function drop1^44^. When models included two-way interactions, also the main terms were included, and if the interaction was not significant, the model was re-run only including the main terms (and these values were included in Table 2). Post-hoc comparisons were then conducted using Tukey tests for significant categorical predictors. We detected no convergence issues. To rule out collinearity, we determined the VIFs^45^, which were minimal (maximum VIFs across all models = 2.28).

## Electronic supplementary material

Below is the link to the electronic supplementary material.

Supplementary Information 1.

Supplementary Video 1.

Supplementary Video 2.

Supplementary Video 3.

Supplementary Video 4.

Supplementary Video 5.

Supplementary Video 6.

Supplementary Video 7.

Supplementary Video 8.

## Data Availability

Will be made available upon publication.
