# Supplementary material for: Dominance style predicts differences in food retrieval strategies
Source: Sci Rep. 2021 Feb 1;11:2726. doi: 10.1038/s41598-021-82198-0 (PMC7851400; doi:10.1038/s41598-021-82198-0)
Supplement: Supplementary file 1 — Supplementary Information 1. [file 41598_2021_82198_MOESM1_ESM.docx]

**SUPPLEMENTARY MATERIAL**

**Methods**

*Subjects.* The study groups included individuals with different sex, age and rank (Table S1). Japanese macaques (JM1) were studied on Koshima Islet, Miyazaki prefecture, Japan (31°27′N, 131°22′E), which is inhabited by two groups of monkeys and several solitary males. In this study, we observed the Main group, which was well habituated and lived in an evergreen broadleaved forest, with access to the beach (for more details on the socio-ecology of the study group, see ^1–3^) . The Barbary macaques (BM3) were studied in Gibraltar (36°08'37.5"N 5°20'36.5"W), in an area with steep cliffs and sparse vegetation. The group lives in a military zone where tourists and inhabitants have no access. The moor macaques (MM4) were studied in the Karaenta forest area, Bantimurung-Bulusaraung National Park, South Sulawesi, Indonesia (5°02’59.4” S, 119°44’13.2” E). The group has been studied for a long time and was well habituated ^4–6^. The monkeys lived in an area with primary forest, in a karst ecosystem.

All study groups mainly foraged and fed on natural food. However, all study groups were also partially provisioned with small quantities of fruit and vegetables by humans other than the experimenters. In particular, JM1 were fed twice a week by a site manager; BM3 in Gibraltar were provided with food on a daily basis by a local NGO; and MM4 were provided with food by occasional local tourists in Sulawesi. As individuals were free to participate in the testing sessions, the number of individuals in each study group does not necessarily correspond to the number of individuals participating in the tasks.

*Materials and procedures.* We conducted our experiments in 2 months for each species, always between October and December (JM1: December 2016; BM3: October and November 2017; MM4: October and November 2018). In order to collect reliable data on individual social behaviour, we conducted behavioural observations for a longer time, on JM1 from November 2016 until June 2017; on BM3 from October 2017 until March 2018; and on MM4 from September 2018 until March 2019. In each group, we conducted hourly instantaneous scans of all group members, recording the spatially closest individual (“nearest neighbour”) of each group member. We conducted 400 group scans in JM1, 47 in BM3, and 364 in MM4.

For each species, we used the Elo method to assess the dominance hierarchy (EloRating package, version 0.43), based on all witnessed dyadic agonistic interactions with a clear winner-loser outcome, recorded via all occurrence sampling during focal and scan sampling ^7^. Dyadic agonistic interactions included aggressions (i.e., threat, chase, bite, lunge), unidirectional agonistic expressions (i.e. open mouth, displacement) and unidirectional submissive behaviours (i.e. make room, bared-teeth display). The values obtained were averaged throughout the study periods, standardized to range from 0 to 1 (with 0 indicating lowest and 1 highest rank), and hereafter simply referred to as Elo-ranks. We included no burn in periods, as Elo-ranks were highly stable throughout the study period (JM1: 0.988; BM3: 0.992; MM4: 0.988), as assessed through visual inspection of the Elo-ranks and with the stab_elo function (which calculates the S index as metric for the overall stability of a hierarchy during the study period, with 0 meaning that the ordering reverses every other day, and 1 that no rank changes occur^8–13).^

Furthermore, to ensure that the study groups really differed in terms of dominance style, we directly assessed the steepness of the dominance hierarchy in each study group ^9^. First, we used the package steepness (version 0.2-2 ^10^) in R to directly assess the steepness of the hierarchy in both species, which is one core measure of macaque dominance styles ^11-12^. We then calculated the steepness as the absolute value of the slope straight line fitted to the normalized David’s scores, obtained on the basis of proportions of wins of dyadic agonistic interactions ^9^. For each species, the individual normalized David’s scores perfectly correlated with the Elo-ranks, as assessed with exact Spearman’s correlations (*p* < 0.001 in all species). As expected, the steepness of the hierarchy was higher in more despotic JM1 (0.280), intermediate in BM3 (0.244) and lowest in less despotic MM4 (0.163). However, steepness is known to decrease with the number of unknown relationships in a group ^13^. As the number of unknown relationships was higher in MM4, we randomly removed dyads with known relationship from the JM1 and BM3 dominance matrix to reach the same proportion of unknown relationships in both species, and averaged the values obtained over 1000 iterations to calculate the adjusted steepness for JM1 (0.255) and BM3 (0.234), which confirmed our classification based on literature (i.e. JM1 are the most despotic species, BM3 intermediate, and MM4 the least despotic one).

**References**

1. Kawai, M. Newly-acquired pre-cultural behavior of the natural troop of Japanese monkeys on Koshima islet. *Primates* **6**, 1–30 (1965).

2. Nakagawa, N., Nakamichi, M. & Sugiura, H. *The Japanese macaques*. (Springer Science & Business Media, 2010).

3. Watanabe, K. Precultural behavior of Japanese macaques: longitudinal studies of the Koshima troops. in *The ethological roots of culture* (eds. Gardner, R., Gardner, B., Chiarelli, B. & Plooiji, F.) 81–94 (Kluwer, 1997).

4. Matsumura, S. Female reproductive cycles and the sexual behavior of moor macaques (*Macaca maurus*) in their natural habitat, South Sulawesi, Indonesia. *Primates* **34**, 99–103 (1993).

5. Matsumura, S. Postconflict affiliative contacts between former opponents among wild moor macaques (*Macaca maurus*). *Am. J. Primatol.* **38**, 211–219 (1996).

6. Okamoto, K. & Matsumura, S. Group fission in Moor macaques (*Macaca maurus*). *Int. J. Primatol.* **22**, 481–493 (2001).

7. Neumann, C. *et al.* Assessing dominance hierarchies: validation and advantages of progressive evaluation with Elo-rating. *Anim. Behav.* **82**, 911–921 (2011).

8. Neumann, C. & Kulik, L. Animal dominance hierarchies by Elo rating. (2020).

9. de Vries, H., Stevens, J. M. & H, V. Measuring and testing the steepness of dominance hierarchies. *Anim. Behav.* **71**, 585–592 (2006).

10. Leiva, D. & de Vries, H. Steepness: testing steepness of dominance hierarchies. (2014).

11. Thierry, B. *et al.* The social repertoire of sulawesi macaques. *Primate Res.* **16**, 203–226 (2020).

12. Balasubramaniam, K. N. *et al.* Hierarchical steepness, counter-aggression, and macaque social style scale. *Am. J. Primatol.* **74**, 915–925 (2012).

13. Klass, K. & Cords, M. Effect of unknown relationships on linearity, steepness and rank ordering of dominance hierarchies: simulation studies based on data from wild monkeys. *Behav. Processes* **88**, 168–176 (2011).

**Table S1. List of the subjects tested in this study, with their sex, age, rank and centrality**.

| **Species** | **Subject** | **Sex** | **Age** | **Rank** |
| --- | --- | --- | --- | --- |
| **Japanese macaques**  **(JM1)** | Beni | Female | Adult | 0.13 |
|  | Betei | Female | Subadult | 0.05 |
|  | Binega | Female | Adult | 0.00 |
|  | Bon | Male | Subadult | 0.14 |
|  | Botan | Female | Juvenile | 0.09 |
|  | Hado | Female | Adult | 0.28 |
|  | Haku | Male | Juvenile | 0.38 |
|  | Hiba | Female | Subadult | 0.20 |
|  | Kanna | Female | Adult | 0.24 |
|  | Kei | Male | Adult | 1.00 |
|  | Keta | Male | Adult | 0.76 |
|  | Kibana | Female | Adult | 0.50 |
|  | Kinoko | Female | Adult | 0.42 |
|  | Kizu | Female | Adult | 0.43 |
|  | Komatsu | Female | Juvenile | 0.32 |
|  | Kote | Male | Adult | 0.48 |
|  | Mekki | Male | Juvenile | 0.14 |
|  | Mikan | Female | Adult | 0.34 |
|  | Minku | Male | Adult | 0.54 |
|  | Mizu | Female | Adult | 0.28 |
|  | Mochi | Female | Juvenile | 0.17 |
|  | Muku | Female | Adult | 0.22 |
|  | Mushi | Male | Adult | 0.51 |
|  | Neji | Male | Adult | 0.40 |
|  | Nire | Female | Adult |  |
|  | Noko | Male | Juvenile | 0.34 |
|  | Noru | Male | Adult |  |
|  | Okapi | Male | Adult | 0.53 |
|  | Okura | Female | Adult | 0.57 |
|  | Omoto | Female | Adult | 0.56 |
|  | Pan | Female | Juvenile | 0.15 |
|  | Pichi | Female | Subadult | 0.09 |
|  | Serori | Female | Adult | 0.25 |
|  | Shida | Male | Juvenile | 0.35 |
|  | Shide | Female | Adult | 0.71 |
|  | Shiira | Male | Subadult | 0.39 |
|  | Shika | Male | Adult | 0.65 |
|  | Sisho | Female | Adult | 0.68 |
|  | Suma | Male | Adult | 0.37 |
|  | Tabu | Female | Subadult | 0.51 |
|  | Toga | Female | Adult | 0.15 |
|  | Tsutsuji | Female | Adult | 0.43 |
|  | Tsuwa | Female | Adult | 0.49 |
|  | Uso | Male | Adult | 0.50 |
|  | Usu | Female | Adult | 0.25 |
|  | Yamu | Female | Adult | 0.66 |
|  | Yashi | Female | Adult | 0.66 |
|  | Yomogi | Male | Subadult | 0.37 |
|  | Yone | Female | Adult | 0.41 |
|  | Yotsuba | Female | Juvenile | 0.25 |
|  | Yumin | Male | Juvenile | 0.17 |
|  | Yuna | Female | Adult | 0.19 |
|  | Yuu | Male | Adult | 0.28 |
| **Barbary macaques**  **(BM3)** | Batmana | Female | Adult | 0.53 |
|  | Chicho | Male | Juvenile | 0.69 |
|  | Colega | Male | Adult | 0.22 |
|  | Grunongibraltar | Male | Adult | 0.67 |
|  | Jefa | Female | Adult | 0.60 |
|  | Legolashijo | Male | Adult | 0.51 |
|  | Legolaspadre | Male | Adult | 0.77 |
|  | Mamabebeenano | Female | Adult | 0.00 |
|  | Mamanoel | Female | Adult | 0.76 |
|  | Mancha | Female | Adult | 0.58 |
|  | Mephisto | Male | Adult | 0.88 |
|  | Nerd | Female | Adult | 0.25 |
|  | Noruega | Female | Adult | 0.48 |
|  | Orejacortada | Female | Adult | 0.37 |
|  | Paul | Male | Adult | 1.00 |
|  | Pedro | Male | Juvenile | 0.39 |
|  | Pendiente | Male | Adult | 0.11 |
|  | Tetas | Female | Adult | 0.06 |
|  | Uniteta | Female | Adult | 0.51 |
| **Moor macaques**  **(MM4)** | Abuabu | Male | Adult | 0.83 |
|  | Adinda | Female | Adult | 0.16 |
|  | Afwan | Male | Juvenile | 0.60 |
|  | Ale | Male | Adult | 0.30 |
|  | Alif | Male | Subadult | 0.40 |
|  | Bb | Female | Adult | 0.15 |
|  | Betty | Female | Adult | 0.04 |
|  | Caca | Female | Adult | 0.34 |
|  | Ciro | Male | Juvenile | 0.15 |
|  | Cri | Female | Adult | 0.04 |
|  | Eli | Female | Adult | 0.41 |
|  | Finny | Female | Adult | 0.11 |
|  | Hantu | Male | Adult | 0.61 |
|  | Hendra | Male | Adult | 0.41 |
|  | Jaya | Male | Adult | 1.00 |
|  | Kopi | Male | Adult | 0.59 |
|  | Lani | Female | Adult | 0.53 |
|  | Lavena | Female | Adult | 0.11 |
|  | Lem | Male | Adult | 0.40 |
|  | Lucia | Female | Subadult | 0.06 |
|  | Moka | Female | Adult | 0.38 |
|  | Momoa | Male | Adult | 0.44 |
|  | Nopi | Female | Adult | 0.24 |
|  | Putri | Female | Adult | 0.12 |
|  | Rissa | Female | Subadult | 0.29 |
|  | Rokko | Male | Adult | 0.22 |
|  | Sahril | Female | Subadult | 0.00 |
|  | Singa | Male | Juvenile | 0.40 |
|  | Sugi | Female | Adult | 0.42 |
|  | Tara | Male | Subadult | 0.51 |
|  | Titi | Female | Adult | 0.28 |
|  | Topi | Male | Adult | 0.73 |
|  | Waps | Male | Adult | 0.72 |

Sex and age were estimated based on visual and behavioural cues in all groups, except for Japanese macaques (for which demographic and life-history data are regularly collected). We then classified as adults all females above 5 and all males above 6 years of age, while juveniles where individuals between 1 and 3 years of age. One corresponds to high rank, and 0 to low rank.
